# Universal time preference

**DOI:** 10.1371/journal.pone.0245692

**Published:** 2021-02-17

**Authors:** Marc Oliver Rieger, Mei Wang, Thorsten Hens

**Affiliations:** 1 Banking and Finance, University of Trier, Trier, Rhineland-Palatinate, Germany; 2 Behavioral Finance, WHU – Otto Beisheim School of Management, Vallendar, Rhineland-Palatinate, Germany; 3 Department of Banking and Finance, University of Zurich, Zurich, Switzerland; 4 Department of Finance, Norwegian School of Economics, Bergen, Western Norway, Norway; 5 Department of Economics, University of Lucerne, Lucerne, Switzerland; Universidad Loyola Andalucia Cordoba, SPAIN

## Abstract

Time preferences are central to human decision making; therefore, a thorough understanding of their international differences is highly relevant. Previous measurements, however, vary widely in their methodology, from questions answered on the Likert scale to lottery-type questions. We show that these different measurements correlate to a large degree and that they have a common factor that can predict a broad spectrum of variables: the countries’ credit ratings, gasoline prices (as a proxy for environmental protection), equity risk premiums, and average years of school attendance. The resulting data on this time preference factor for *N* = 117 countries and regions will be highly useful for further research. Our aggregation method is applicable to merge cross-cultural studies that measure the same latent construct with different methodologies.

## Introduction

What is time? This is a fascinating question to almost every human being. Einstein reminded us that “the distinction between the past, present, and future is only a stubbornly persistent illusion.” In his book “Time Wars”, Jeremy Rifkin noted that “Every culture has its own unique set of temporal fingerprints. To know a people is to know the time values they live by.” Rifkin observes that “All of our perception of self and world is mediated by the way we imagine, explain, use, and implement time” [1]. Each culture has its own perception, preference, and social norms regarding time [2]. Is there a way to measure this cultural variation of “temporal fingerprints”?

Different societies have dramatically different attitudes and perceptions towards time [2]. For example, even within the same country, Romani people from Southern Spain can adopt “faster” life-history strategies than their non-Romani neighbors, as shown in [3]. Our study focuses, however, on country-differences, as previous literature has shown that, both theoretically and empirically, the cross-cultural difference in time preference is related to national saving, investment behavior, economic growth, among many others [4, 5]. It is therefore important to reliably measure the cross-cultural differences in societal time orientation.

In this paper, we compare and integrate several important previous studies on cross-cultural differences in time orientation. These studies use different methods from different disciplines, from discounting factors in economic models [6], field study from social psychologists [2], to survey on cultural values in sociological studies [4]. All these studies compare different countries with regards to time-related perspective, but to date, there is no systematic study to compare the reliability and validity of these cross-country studies. Our paper aims to test the cross-study reliability of these measures and compose a common factor underlying these measurements, encompassing as many countries as possible. The good news is that we find converging evidence of cultural variations of such “temporal fingerprints”, and we illustrate the predictive power of our composite index on various behaviors at the country level, ranging from savings to environmental protection.

Scientists have developed many different methods to trace these “temporal fingerprints”, or time-related preference, norms, and behavior. For example, economists typically measure the discount rate between current and future payoffs through field and experimental studies, either inferred from consumers’ choices or elicited with stylized intertemporal prospects [7, 8]. See [9] for a review on the measurement of time preferences by economists. Sociologists ask statements related to time orientation, e.g., the importance of thrift and tradition [4]. Social psychologists observe actual behavior in everyday situations, e.g., the average speediness of salespersons in standardized tasks [2]. A priori, it is not clear whether these methods measure the same concept–an important and unfortunately often overlooked issue [10]–although occasionally, relations between different methodologies have been found [6].

In this article, we collect information from several large-scale international studies on time orientations and time preferences that used very different methodologies. We show that there is one clear underlying factor that can be extracted via principal component analysis. Using an appropriate statistical method, we can estimate this factor for a large number of countries and regions (*N* = 117). The factor is then shown to have high external validity: it is related to several variables that have previously been suggested to be influenced by time preferences.

Therefore, in the age of a replication crisis, following [11] our results provide good news for researchers in the field of culture and time: the previous studies reveal one common factor related to a nondomain-specific “time value” that can predict a variety of general country differences at the aggregate level. We also demonstrate a useful method for cross-cultural social scientists to test the convergence of interdisciplinary cross-national empirical studies at a relatively low cost.

## Data and methodology

### Methodology

The key difficulty in defining a universal time preference factor is that the various measurements of time preferences differ in their ways of measurement, their order of magnitude, and the number of countries they cover. In the following, we will describe our method that can circumvent these problems to arrive at a reasonable definition of a common factor in time preferences, a universal time preference “UP-time”.

The ten measured variables of time preferences used as starting points for our study have been elicited using very different definitions and estimation methods (Table 1). One of the studies used field data [2], all others survey data. None of the questions in these studies were incentivized.

**Table 1:** Overview of the international studies on time preferences.

Study	Variable	Method
INTRA [6]	Patience (% of “patient” subjects)	Binary payment choice (1 month)
	Delta (median)	Certainty equivalence questions on payoffs in 1 year and 10 years
	Beta (median)	
Falk et al. [18]	Patience (average)	Certainty equivalence question and self-assessed patience (weighted average of both)
Breuer et al. [17]	Beta (average)	Certainty equivalence questions on payoffs in 1 year and 10 years
	Delta (average)	
Levine et al. [2]	Pace of life (average of the Z-scores of each item)	Based on average walking speed, accuracy of clocks, postal service speed
World Value Survey [25]	Long-term orientation (LTO) (average)	Likert-scale questions about the importance of thrift, tradition, etc.
GLOBE [29]	Future-orientation, societal practices (FOSP) (average)	Likert-scale questions about time orientation in behavior
	Future-orientation, societal values (FOSV) (average)	Likert-scale questions about time orientation in values

We used these six studies with ten measurements of time preference for our analysis. The methodology of these studies varies largely. Aggregation methods are as in the original studies and noted in parentheses.

In a first step, we need to unify their scaling to make them comparable. To this end we use the country level averages of the datasets and compute for each variable its Z-score, i.e. we standardize the data`s country mean to zero and their country variance to one. We also arrange the signs such that more “patient” values have a positive sign and more “impatient” values a negative.

To determine a common time preference factor, we use a principal component analysis (PCA) on the Z-scores of the ten variables. We apply this method to extract the main factor, i.e. the factor with the largest eigenvalue. We do not rotate the resulting factor matrix (as this wouldn’t be meaningful for a single factor), and we replace missing data by means.

The PCA shows that time preferences indeed share one strong common factor that can explain around one third of the total country variation.

Since some studies provided more than one measurement to our dataset, we also conducted a PCA on a subset of six measurements, using only one measurement per study to avoid the overrepresentation of certain studies. For each of these studies, we selected the measurement that has either been demonstrated to capture the behavioral aspects of time preferences better in previous studies [6] and if that was unknown, the measurement that had a higher correlation with the other variables. For the six variables selected in this way, we apply the same method as described above, converting them to Z-scores and using a PCA, to define a single factor as the factor with the largest eigenvalue.

In both cases, starting with all ten or with the selected six variables, we arrive at weights *w*_*f*_ for the variables *f* = 1,…,*F* with *F* = 10 or *F* = 6, respectively.

We then compute non-normalized weights $\overset{-}{w_{f,c}}$ for each country *c* and each variable *f* according to the formula

$$\overset{-}{w_{f,c}}=\left\{ \begin{array}{cc} w_{f}, & if\;data\;for\;that\;country\;and\;variable\;exists,\\ 0, & if\;no\;such\;data\;exists, \end{array}\right.$$

where *w*_*f*_ denotes the PCA weights and *F* is the total number of variables used (6 or 10, in the two setups that we use). We then normalize these weights such that their sum is one for each country:

$$w_{f,c}\;=\;\frac{\overset{-}{w_{f,c}}}{\sum_{i\;=\;1}^{F}\overset{-}{w_{\dot{i},c}}}$$

These normalized weights *w*_*f*,*c*_ are then used to compute the universal preference of time (UP time) variables *UPT*^*c*^ for each country *c* by simply taking the weighted average over the time preference variables for this country:

Let *Z*_*f*,*c*_ be the Z-score of the time preference variable *f* in country *c*, then we define

$${UPT}^{c}≔\sum_{f\;=\;1}^{F}w_{f,c}Z_{f,c.}$$

If a particular time preference variable for a specific country is not defined, we simply set *Z*_*f*,_*c = 0*, *since* the corresponding entry for the weight *w*_*f*,*c*_ is zero, and therefore this summand is simply omitted.

Let us explain our method with an example:

In this example, for simplicity, we have only three countries (*c* = 1,2,3) and only three time preference variables (*f* = 1,2,3) where the first variable assigns higher values to a more “patient” country and the second and third one lower values. The measurements for the three countries are as follows:

$$\begin{array}{cccc} & \text{Time}\;\text{preference}\;1 & \text{Time}\;\text{preference}\;2 & \text{Time}\;\text{preference}\;3\\ \text{Country}\;1 & 10 & -0.5 & 100\\ \text{Country}\;2 & 8 & 0.0 & 55\\ \text{Country}\;3 & 6 & & 50 \end{array}$$

We see that these measurements pose three challenges: first, as we have already mentioned, the countries’ directions differ. Second, their magnitudes differ substantially as well. Third, country 3 has only data for two variables instead of three.

To solve the first two issues, we invert the second and third scale and compute for each of them their respective Z-values. This leads to the following result (notice that for each variable, now the mean is zero and the variance and standard deviation are one):

$$\begin{array}{cccc} & \text{Z-value}\;\text{of}\;\text{Time}\;\text{preference}\;1 & \begin{array}{c} \text{Z-value}\;\text{of}\;\text{Time}\;\text{preference}\;2 \end{array} & \begin{array}{c} \text{Z-value}\;\text{of}\;\text{Time}\;\text{preference}\;3 \end{array}\\ \text{Country}\;1 & 1 & 0.707 & 1.150\\ \text{Country}\;2 & 0 & -0.707 & -0.484\\ \text{Country}\;3 & -1 & & -0.666 \end{array}$$

Now we apply a principal component analysis (PCA) on these values, replacing the missing value with its average. We obtain the following factor loadings (calculation can be done in any statistics software; we used SPSS):

$$\begin{array}{ccc} & \text{First}\;\text{factor}\;\text{of}\;\text{PCA}: & \\ \text{Z-value}\;\text{of}\;\text{Time}\;\text{preference}\;1 & 0.888 & \text{defined}\;\text{as}\;w_{1}\\ \text{Z-value}\;\text{of}\;\text{Time}\;\text{preference}\;2 & 0.842 & \text{defined}\;\text{as}\;w_{2}\\ \text{Z-value}\;\text{of}\;\text{Time}\;\text{preference}\;3 & 0.999 & \text{defined}\;\text{as}\;w_{3} \end{array}$$

The non-normalized weights are then simply these factors or zero (if there is no data for a particular variable and a particular country):

$$\begin{array}{cccc} \bar{w_{f,c}} & f=1 & \begin{array}{c} f=2 \end{array} & \begin{array}{c} f=3 \end{array}\\ c=1 & 0.888 & 0.842 & 0.999\\ c=2 & 0.888 & 0.842 & 0.999\\ c=3 & 0.888 & 0 & 0.999 \end{array}$$

Now we compute the weights *w*_*f*,*c*_ using the formula previously defined:

$$\begin{array}{cccc} w_{f,c} & f=1 & \begin{array}{c} f=2 \end{array} & \begin{array}{c} f=3 \end{array}\\ c=1 & 0.888/(0.888+0.842+0.999) & 0.842/(0.888+0.842+0.999) & 0.999/(0.888+0.842+0.999)\\ c=2 & 0.888/(0.888+0.842+0.999) & 0.842/(0.888+0.842+0.999) & 0.999/(0.888+0.842+0.999)\\ c=3 & 0.888/(0.888+0.999) & 0 & 0.999/(0.888+0.999) \end{array}$$

We see here how to deal with the missing data for country 3: the respective weight *w*_*2*,*3*_ is zero and the corresponding weights for the other factors for this country are correspondingly larger. This ensures that the sum over all weights *w*_*f*,*c*_ for each country is still one since otherwise we would artificially decrease the absolute value of *UPT*^*c*^ for countries with fewer data points.

We compute the formulas in the above table and then apply the formula for *UPT*, e.g.:

$$UPT^{1}=w_{1,1}\cdot Z_{1,1}+w_{2,1}\cdot Z_{2,1}+w_{3,1}\cdot Z_{3,1}=0.325\cdot 1+0.309\cdot 0.707+0.366\cdot 1.149.$$

In this way we obtain the following result:

$$\begin{array}{ccccc} w_{f,c} & f=1 & f=2 & f=3 & UPT^{c}\\ c=1 & 0.325 & 0.309 & 0.366 & 0.964\\ c=2 & 0.325 & 0.309 & 0.366 & -0.395\\ c=3 & 0.471 & 0.000 & 0.529 & -0.823 \end{array}$$

This concludes our example (which was of course chosen for instructive purposes only, as three countries are too few to allow for a meaningful application of our method).

We finally define the country weights

$$w^{c}≔\frac{\sum_{f=1}^{F}{\overset{-}{w}}_{f,c\;}∙\delta_{n,f}}{\sum_{f=1}^{F}{\overset{-}{w}}_{f,c\;}}$$

where *δ*_*n*,*f*_ equals one if the measurement of the variable *f* exists for country *n* and zero otherwise. These country weights provide a measure for the validity of the UP-time variable in a country *c*: If data are only available for very few time discounting measurements with low weights, this variable will be close to zero. If, on the other hand, data is available for all measurements, the weight will be one. You can think about *w*^*c*^ as a quality measurement of the data computed for one country. These weights will be used in the weighted regressions of Table 5, and should also be used when applying the UP-time data in future studies.

In our example, the country weights for countries 1 and 3 are indeed one (the weight of country 1, e.g., can be computed as (0.888 + 0.842 + 0.999)/(0.888 + 0.842 + 0.999) = 1.000). The weight of country 2, however, is only (0.888 + 0.999)/(0.888 + 0.842 + 0.999) = 0.691. Thus, country 2 will be less important in further analysis as its data quality is not as good as the one from country 1 and country 3. In this way, outliers in a few measurements of one country will not have too much impact on further analysis.

### Data and related variables

The data sources for all variables that we use in our study (previous time preference measurements as well as the various other variables used in Table 5) are specified in Table B in S1 File. In the following, we describe these data in more detail.

#### Equity risk premium

Equity risk premium refers to the excess return of stocks over bonds, which is relatively difficult to measure. By definition, we are looking for long-term differences in stock market and bond returns, and the time horizon for a very good estimate can easily be more than 100 years. In most countries, however, stock markets do not exist that long or at least there is no data readily available for them. Using stock market indices as a proxy is suboptimal as it might underestimate the effects of small stocks that might change the long-term return of the whole stock market substantially.

A set of historical equity risk premia has first been collected in [12] but only for 17 countries. A thorough statistical analysis comparing these data with our UP-time variable is therefore not possible, but at least we find a substantial negative correlation (depending on the model, around –40%). A much more comprehensive dataset using a survey methodology has been provided by [13]. They proxy the equity risk premium by an average of expert estimates from the respective country. In total, 74 countries for which we have UP-time data also are also covered by [13]. This overlap is substantial enough to apply the required statistical analysis.

One possible explanation for the equity risk premium [14] is that the returns from equity are more procyclical to consumption growth than the interest earned on bonds. On average households have a decreasing marginal utility from consumption implying that in their inter-temporal consumption optimization they require higher compensation for equity than for bonds. Moreover, the more impatient households are the shorter is their planning horizon [15]. Since in the short-term, equity returns are riskier than in the long-term, the more impatient households are the higher is the risk premium they require for holding equity. Such explanations imply that cultural differences can exist as well. Indeed, differences between countries have been found and associated with differences in (average) behavioural preferences [16]. Among these behavioral factors are time preferences, thus it seems natural to test for a relation between the equity premium and the UP-time variable.

#### Average years in school (schooling)

This variable measures how many years a person in a country on average spends in total for education (school and university). An average high school graduate from the US as well as an average French graduate with a baccalauréat, both without further education, would have spent 12 years, while a person with a Ph.D. degree might have spent 20 years and others, particularly in poorer countries, might have obtained only a few years of elementary education.

Although this variable is of course not capable of measuring all quality differences between school systems (10 years of education in one country might be better than 12 years in another, and the additional value of education beyond a certain point can also be questioned), the average time can serve at least as a proxy for the importance that a society places on education–on the individual level of families and students that use the education system, but also on the societal level that provides schools and universities. Of course, on both levels, limitations arise due to economic constraints, thus we have to control for them in our analysis.

#### Human Development Index (HDI)

The human development index encompasses several factors to assess the overall development of a country by considering the factors of life expectancy, education, and income.

#### Credit rating

The credit rating of a country (or more precisely: the credit rank of its long-term sovereign bonds) is measured by rating agencies. We use data from the three biggest rating agencies, Standard & Poor’s, Fitch and Moodys, converted into a numerical scale, and average them. (Differences between the ratings of different agencies for one country are usually very small.) We use the values of July 31, 2020, but although these values tend to change over time, the relative differences between countries are fairly stable.

#### Gasoline price

We define this variable as the ratio of the gas price in a country and the world average (not weighted). As differences in gasoline prices between countries are mostly driven by the amount of taxes, and the taxes are motivated at least in parts by environmental protection reasons (e.g., reduction of greenhouse gas emissions), this serves as a good proxy for the importance of environmental politics in a country.

#### Resilience index

The FM Global Resilience index helps to assess the capacity of different countries to withstand and recover from disruption. The index consists of 12 resilience drivers, subdivided into aspects of business, risk quality, and the supply chain. Factors are political risk, oil intensity, urbanization rate, exposure to natural hazards, natural hazard risk quality, fire risk quality, inherent cyber risk, control of corruption, quality of infrastructure, local supplier quality, supply chain visibility, and productivity. The resilience to many of these factors can be improved by a far-sighted state, but in many cases, this requires long-term efforts which makes a connection with time preferences plausible.

## Results

### Cross-study reliability

We use data on time orientation and time preferences from six systematic international comparison studies (Table 1). Although the methodology of these studies varies widely, we find high correlations between them (Table 2). A reliability analysis yields a Cronbach’s alpha of 0.893 (0.792 when taking out the two studies with the lowest number of countries). Both results suggest that there is a common factor underlying country variations in time preferences.

**Table 2:** Correlations of the ten time-preference variables.

	INTRA			Falk et al.	Breuer et al.		Levine et al.	WVS	GLOBE	
	Patience	Delta	Beta	Patience	Beta	Delta	Pace	LTO	FOSP	FOSV
Patience	1	.365**	.445**	.632**	0.657	.791*	.472*	.290*	.461**	-.486**
		0.007	0.001	0	0.109	0.034	0.027	0.039	0.004	0.002
	53	53	53	40	7	7	22	51	38	38
Delta	.365**	1	.591**	.380*	-0.004	-0.072	0.369	-0.052	0.263	-0.147
	0.007		0	0.016	0.994	0.877	0.091	0.719	0.111	0.377
	53	53	53	40	7	7	22	51	38	38
Beta	.445**	.591**	1	.535**	-0.114	-0.024	0.344	-0.169	.379*	-0.115
	0.001	0		0	0.808	0.96	0.117	0.237	0.019	0.492
	53	53	53	40	7	7	22	51	38	38
Patience	.632**	.380*	.535**	1	-0.075	0.4	.597**	.355**	.678**	-.540**
	0	0.016	0		0.86	0.326	0.003	0.005	0	0
	40	40	40	76	8	8	23	61	43	43
Beta	0.657	-0.004	-0.114	-0.075	1	.836**	0.141	-0.027	.779*	-0.123
	0.109	0.994	0.808	0.86		0.005	0.822	0.944	0.013	0.753
	7	7	7	8	9	9	5	9	9	9
Delta	.791*	-0.072	-0.024	0.40	.836**	1	0.010	0.451	.756*	-0.411
	0.034	0.877	0.96	0.326	0.005		0.987	0.223	0.018	0.272
	7	7	7	8	9	9	5	9	9	9
Pace	.472*	0.369	0.344	.597**	0.141	0.01	1	0.346	0.266	-0.279
	0.027	0.091	0.117	0.003	0.822	0.987		0.077	0.199	0.177
	22	22	22	23	5	5	30	27	25	25
LTO	.290*	-0.052	-0.169	.355**	-0.027	0.451	0.346	1	0.227	-.426**
	0.039	0.719	0.237	0.005	0.944	0.223	0.077		0.113	0.002
	51	51	51	61	9	9	27	95	50	50
FOSP	.461**	0.263	.379*	.678**	.779*	.756*	0.266	0.227	1	-.266*
	0.004	0.111	0.019	0	0.013	0.018	0.199	0.113		0.044
	38	38	38	43	9	9	25	50	58	58
FOSV	-.486**	-0.147	-0.115	-.540**	-0.123	-0.411	-0.279	-.426**	-.266*	1
	0.002	0.377	0.492	0	0.753	0.272	0.177	0.002	0.044	
	38	38	38	43	9	9	25	50	58	58

** Correlation is significant at the 1% level,

* significant at the 5% level.

For each pair of measurements, the Pearson correlation coefficient, the p-value and the number of countries for which both data sets provide cases are displayed.

We estimate the common factor of time preferences using principal component analysis (PCA) for the set of all studies and (as robustness test) for a subset of the studies to avoid the overrepresentation of a single study. The factor loadings are shown in Table 3. Then, we use these factor loadings to estimate a universal preference variable for the set of all *N* = 117 countries and regions in our data. (See the methodology section for details on the estimation procedure). We call the resulting factor “universal preferences for time” (short: UP time).

**Table 3:** Weights of the Principal Component Analysis (PCA).

Study	Variable	Weights (PCA)	
INTRA	Patience	0.741	0.699
	Delta	0.488	
	Beta	0.554	
GPS	Patience	0.724	0.780
Breuer et al.	Beta	0.381	0.329
	Delta	0.449	
Levine et al.	Pace	0.460	0.554
WVS	LTO	0.322	0.442
GLOBE	FOSP	0.700	0.743
	FOSV	0.563	
Total variance explained		30.9%	37.7%

In the first model, all ten measurements were used; in the second model, only six were chosen to avoid overrepresenting studies with more than one measurement.

Since we carry out the first analysis with ten measurements and the second with a subset of them, we have two resulting variants of the UP time variable. The resulting country-level data are shown in Table A in S1 File, together with weights that specify the reliability of each particular measurement (see the methods section for details).

The correlations of UP time with previous studies are high and statistically significant (except the data from [17], which measured only nine countries), see Table 4. Given that the variable is computed using the data from these studies, this is not too surprising. The degree of correlation of the standard UP time variable with the single study variables (in all cases exceeding 0.6) and its significance (mostly p<0.001) is still noteworthy and again underlines the existence of a common factor in time preference measurements.

**Table 4:** Correlations of the UP time variables with previous measurements.

	UP Time 10	UP Time 6
Patience	.824**	.835**
(INTRA)	<0.001	<0.001
	53	53
Delta	.612**	.342*
(INTRA)	<0.001	0.012
	53	53
Beta	.690**	.448**
(INTRA)	<0.001	0.001
	53	53
Patience	.890**	.897**
(GPS)	<0.001	<0.001
	76	76
Beta	0.647	0.646
(Breuer et al.)	0.06	0.06
	9	9
Delta	.809**	.817**
(Breuer et al.)	0.008	0.007
	9	9
Pace	-.716**	-.740**
(Levine et al.)	<0.001	<0.001
	30	30
LTO	.602**	.685**
(WVS)	<0.001	<0.001
	95	95
FOSP	.742**	.809**
(Globe)	<0.001	<0.001
	58	58
FOSV	-.673**	-.543**
(Globe)	<0.001	<0.001
	58	58

** Correlation is significant at the 1% level (2-tailed).

* Correlation is significant at the 5% level (2-tailed).

Pearson correlations, p-values and number of common country data entries are displayed.

**Fig 1:**
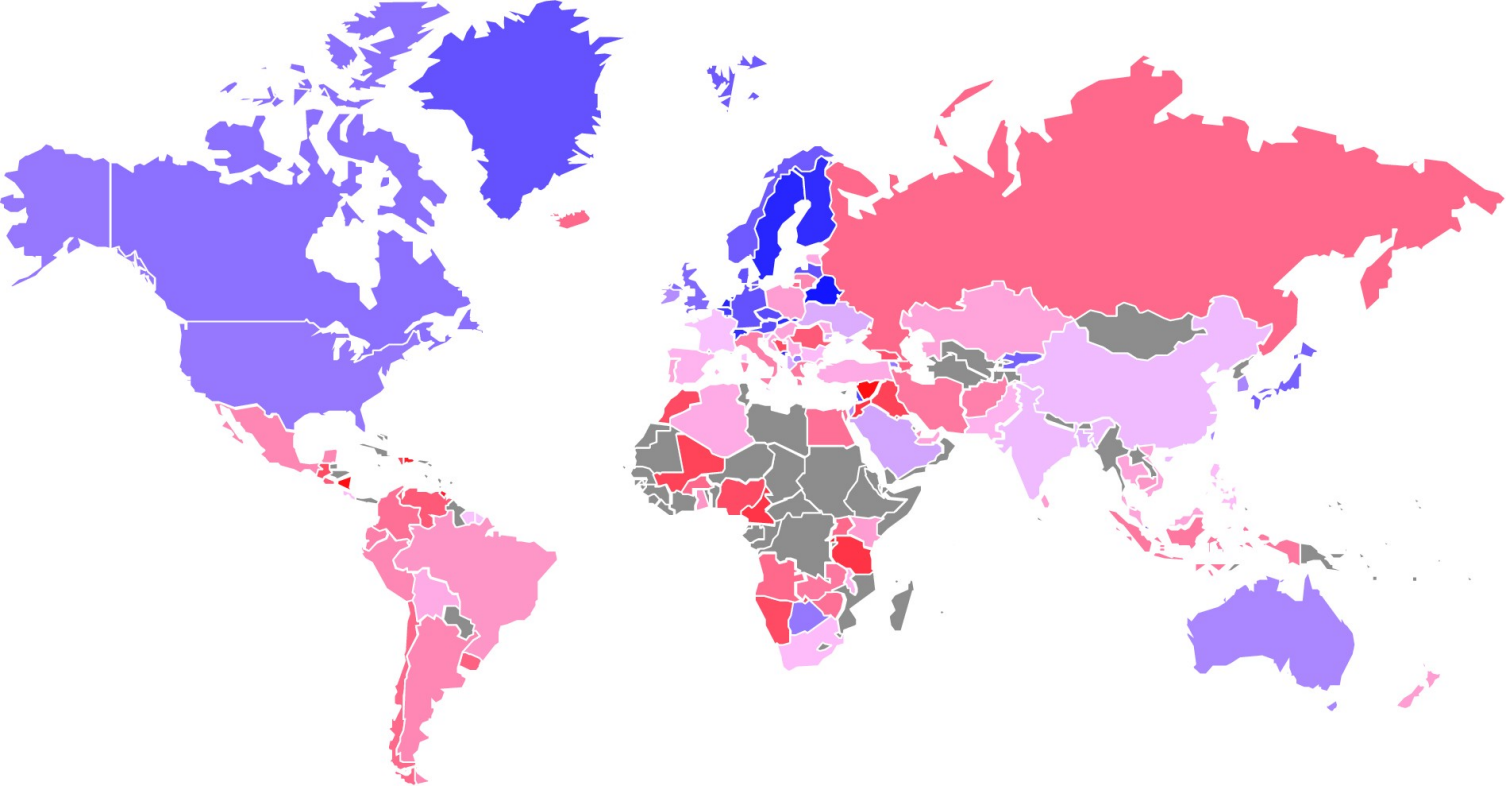
World map of time preferences. Countries with preferences for more immediate rewards are colored in red, countries that emphasize future rewards more are marked in blue, gray colors indicate missing data. Reprinted from https://www.clearlyandsimply.com/clearly_and_simply/2009/06/choropleth-maps-with-excel.html under a CC BY license, with permission from Robert Mundigl (clearlyandsimply), original copyright 2009.

Fig 1 provides a world map where the UP time variable (estimated from the full set of studies) is color-coded. While there seem to be some outliers (usually associated with a very low weight, which is not shown in the map), a general pattern emerges, with the Anglo-American countries and Central and Northern Europe showing generally very high numbers (corresponding to “patient” time discounting), Southern and Eastern Asian countries showing medium-high numbers, and South American, Southern European, and African countries showing low numbers. Countries in the Middle East and Eastern Europe have heterogeneous values. This already suggests that between-country variation is neither random nor simply determined by interest rates nor, more generally, economic stability. Instead, cultural factors seem to play a role as well, following earlier results.

### External validity

Estimating a large international dataset on time preferences is only meaningful if the data describe or predict actual real-life phenomena. To test this, we used country-level variables from very different fields where some have been previously applied to test international measurements of time preferences [6, 15, 16, 18–21]. Details of the estimation and data sources are provided in the methods section:

- Equity risk premium, i.e. the excess return of stocks over bonds.
- Level of education (proxied by the average years of school attendance),
- The overall development of the countries (proxied by the human development index),
- Credit rating (estimating a country’s risk to default),
- Environmental protection (proxied by the average price for gas since this price depends mostly on taxation that is motivated by environmental concerns),
- Resilience (a measure to capture the capacity of a country’s business environment to withstand disruption and recover strongly, should a disruption occur. It compiles 12 economic, risk quality, and supply chain drivers).

In all of these cases, time preferences should play a predictive role: there is always a tradeoff between short-term costs (decreasing spending, driving less, studying longer, etc.) versus a long-term benefit (e.g., less debt, clean environment and less global warming, better job perspectives) [20, 22–24]. However, we should note that reverse causality is likely as well. For example, although patience can increase wealth accumulation, wealthier countries can also afford to be more patient because the future is more stable and more predictable.

**Fig 2:**
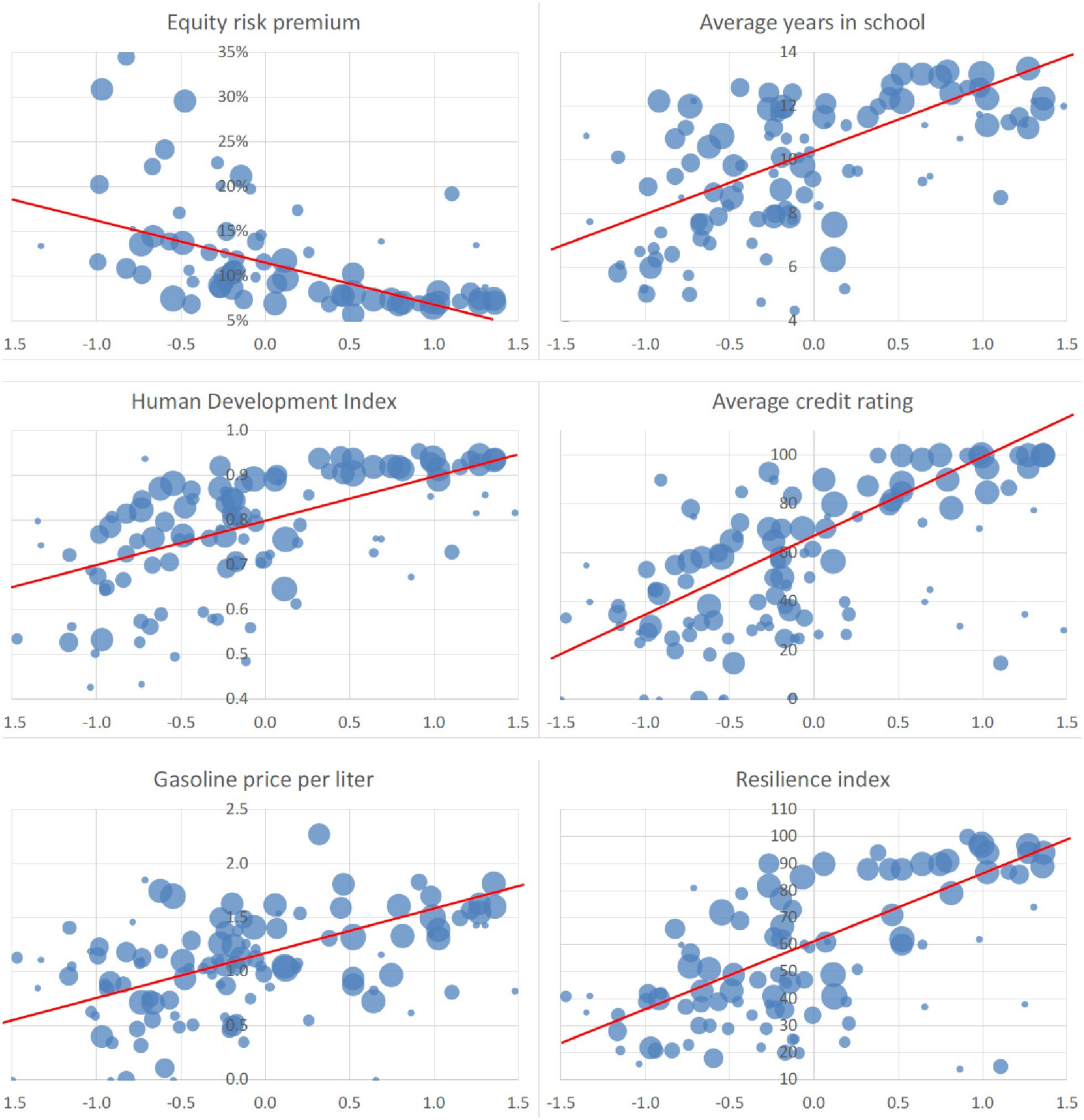
Bubble plots of various variables and universal preferences for time (UP time, on the x-axis). In most cases, we can see a clear dependence. The sizes of the bubbles correspond to the weight of the country data. Weighted regression lines in red.

Indeed, we find in most of these cases a strong relation of these variables to UP time, as the bubble plots (Fig 2) indicate (where the bubble sizes correspond to the respective country weights).

A correlation analysis is, of course, only the first step: certain economic factors influence all of these parameters: wealthier countries have it easier, e.g., to protect the environment or to improve education. There are also obvious interrelations between time preferences and wealth because causality might work in both ways here: more “patient” countries might become richer, but richer, more stable countries can more easily be “patient”. Since this effect has already been demonstrated in previous work [6, 25]. we need to control for country wealth (proxied by GDP per capita).

Other economic reasons might lead to steeper time discounting, in particular economic instability and high-interest rates. We therefore also control for the volatility of the GDP growth (measured over the past 20 years), the interest rate, and the credit spread of government bonds.

For each of the eight variables above, we, therefore, conduct a regression analysis, controlling for each of these four factors. To avoid collinearity issues, we usually control for one variable at a time. We use UP10 as the explanatory variable, but identical regressions with UP6 lead basically to the same significance results (provided on request). As weighting factors, we use the aforementioned weights (see Table A in S1 File). We also calculate the difference between the adjusted R^2^ when including UP10 into the regression versus the same regression *without* UP10 to illustrate the additional explanatory power of time discounting.

The results of the regressions are presented in Table 5. We see that in nearly all cases, the UP time variables have a significant and often substantial predictive power. Only the p-value in the case of the gas price is above 0.05 in one model, while the p-values for credit rating and resilience are below 0.1% for all models. The predictive power is also substantial: using only UP time and GDP per capita, it is possible to explain approximately 50% of the variation in the education variable and more than 70% of the credit rating and resilience.

**Table 5:** Regression analysis for external validity.

**Equity risk premium**	Model 1	Model 2	Model 3	Model 4	**Level of Education**	Model 1	Model 2	Model 3	Model 4
UP10	-0.35* (-2.62)	-0.48*** (-5.07)	-0.26*** (-4.26)	-0.19** (-2.92)	UP10	0.28** (2.91)	0.65*** (8.36)	0.55*** (6.61)	0.51*** (4.77)
GDP/cap	-0.31* (-2.34)				GDP/cap	0.47*** (4.83)			
Vola GDP growth		0.32*** (3.39)			Vola GDP growth		0.14* (1.79)		
Interest rate			0.75*** (12.14)		Interest rate			-0.14 (-1.66)	
Credit spread gov. bonds				0.79*** (11.94)	Credit spread				-0.21* (-1.91)
N	70	72	71	57	N	108	111	107	68
adj. R^2^	0.36	0.40	0.78	0.81	adj. R^2^	0.48	0.38	0.38	0.39
additional R^2^	0.05	0.21	0.05	0.03	additional R^2^	0.04	0.39	0.25	0.20
**HDI**	Model 1	Model 2	Model 3	Model 4	**Credit Rating**	Model 1	Model 2	Model 3	Model 4
UP10	0.24** (2.84)	0.67*** (8.96)	0.55*** (7.2)	0.5*** (5.16)	UP10	0.32*** (4.13)	0.68*** (9.89)	0.55*** (8.72)	0.5*** (6.46)
GDP/cap	0.60*** (7.12)				GDP/cap	0.58*** (7.61)			
Vola GDP growth		0.05 (0.66)			Vola GDP growth		-0.13* (-1.91)		
Interest rate			-0.25*** (-3.28)		Interest rate			-0.4*** (-6.34)	
Credit spread gov. bonds				-0.33*** (-3.40)	Credit spread gov. bonds				-0.47*** (-6.00)
N	108	111	107	68	N	105	107	105	69
adj. R^2^	0.61	0.42	0.48	0.50	adj. R^2^	0.69	0.53	0.65	0.68
additional R^2^	0.03	0.41	0.25	0.19	additional R^2^	0.05	0.43	0.25	0.19
**Gasoline price**	Model 1	Model 2	Model 3	Model 4	**Resilience index**	Model 1	Model 2	Model 3	Model 4
UP10	0.20* (1.76)	0.45*** (5.14)	0.33*** (3.79)	0.35** (2.98)	UP10	0.25*** (3.4)	0.7*** (9.5)	0.58*** (8.16)	0.52*** (6.13)
GDP/cap	0.40*** (3.57)				GDP/cap	0.66*** (9.01)			
Vola GDP growth		-0.11 (-1.21)			Vola GDP growth		-0.05 (-0.71)		
Interest rate			-0.36*** (-4.08)		Interest rate			-0.32*** (-4.54)	
Credit spread gov. bonds				-0.27* (-2.33)	Credit spread gov. bonds				-0.41*** (-4.86)
N	107	109	106	68	N	100	101	100	68
adj. R^2^	0.30	0.23	0.32	0.27	adj. R^2^	0.72	0.50	0.58	0.63
additional R^2^	0.01	0.18	0.09	0.09	additional R^2^	0.03	0.45	0.28	0.20

** Correlation is significant at the 1% level,

* significant at the 5% level.

Weighted regressions of the eight variables (equity risk premium, schooling, HDI, credit rating, environmental protection and resilience) on UP10 with various control variables. T-values are in parentheses.

These examples demonstrate that our time preference variable, UP time, has a high external validity across a broad range of applications. It also shows that this is not because the variable is simply a proxy for economic wealth, as we have controlled for this variable in the above regressions.

## Discussion

Our paper has made three important contributions: (1) we cross-validate the measurement of time orientation by comparing studies with different methods (questions on monetary tradeoff, survey questions on general attitudes, and field study on societal behavior) from different disciplines (economics, psychology, and sociology); (2) we proposed a method to standardize and extract common factor from cross-country studies with different instruments and scales. This methodology can be applied to other domains to synthesize international data. (3) we provide country-level measurement to proxy time orientation for a large number of countries (N = 117). This dataset can be used for future studies related to cross-country study on intertemporal decisions.

We have seen that different measurements of time preferences on the country level have a unique underlying factor, a “temporal fingerprint”, as we mentioned at the beginning of this article. This resonates with John Rae’s conjecture that countries differ in their “effective desire of accumulation,” a sociological and psychological factor, which in turn influences the production activity and national wealth [26]. Combining those previous measurements, this factor can be estimated for a large number of countries. The factor has good external validity and can predict various variables connected to time preferences, making it highly useful as a foundation for future studies in this field. A strong advantage of the new dataset is that it contains a large number of countries, which allows us to control for more country-level variables than was previously possible.

Our approach certainly hinges on high-quality data from which we can build up our dataset. While none of the original studies used monetary incentives for the time preference measurements, several studies verified that monetary incentives do not significantly affect time preference questions [17, 27, 28]. While we have seen that all datasets reflect a common concept of time preferences, some are better than others in capturing this concept. We consider these differences by using different weights for each study (obtained from a principal component analysis). There are also some countries for which the coverage in previous studies is low or that have not yet been covered at all. This leads to missing data points and a few outliers. Hence, there is a need for further international studies on time discounting to be included in our dataset.

For the abovementioned reasons, it would not be appropriate to derive a “country ranking” of time preferences from our data since countries covered in only one study will tend to have more extreme evaluations. We recommend instead using the data only as a basis for statistical analysis. If possible, data should be weighted by our weighting parameters. Since the UP10 and UP6 data do not show large differences regarding their external validity, we recommend using the UP10 data since it is derived from a larger set of measurements.

## Supporting information

S1 Data (XLSX)

S1 File (DOCX)
