# Supplementary material for: Universal time preference
Source: PLoS One. 2021 Feb 17;16(2):e0245692. doi: 10.1371/journal.pone.0245692 (PMC7888607; doi:10.1371/journal.pone.0245692)
Supplement: S1 File — (DOCX) [file pone.0245692.s002.docx]

**Table A. List of the time preference country data in our study.** The time preference variables are denoted by UP (10) for the universal preferences of time based on all ten measurements and UP (6) for that based only on the selection of six measurements. The columns labeled “weights” signify the reliability of each data entry and should be used in regression analysis as weights.

| Country/region | UP10 | weights | UP6 | weights |  | Country/region | UP10 | weights | UP6 | weights |
| --- | --- | --- | --- | --- | --- | --- | --- | --- | --- | --- |
| Afghanistan | -0.54 | 0.13 | -0.54 | 0.22 |  | Guatemala | -0.94 | 0.37 | -0.97 | 0.43 |
| Africa East | -0.54 | 0.06 | -0.54 | 0.12 |  | Haiti | -1.01 | 0.13 | -1.01 | 0.22 |
| Africa West | -1.50 | 0.06 | -1.50 | 0.12 |  | Hong Kong | 0.32 | 0.71 | 0.65 | 0.69 |
| Albania | 0.21 | 0.29 | 0.31 | 0.33 |  | Hungary | -0.19 | 0.85 | -0.36 | 0.91 |
| Algeria | -0.13 | 0.19 | -0.18 | 0.34 |  | Iceland | -0.71 | 0.06 | -0.71 | 0.12 |
| Angola | -0.74 | 0.33 | -0.58 | 0.20 |  | India | 0.11 | 0.91 | 0.17 | 0.84 |
| Arab countries | -0.92 | 0.06 | -0.92 | 0.12 |  | Indonesia | -0.57 | 0.51 | -0.58 | 0.71 |
| Argentina | -0.47 | 0.76 | -0.79 | 0.75 |  | Iran | -0.59 | 0.58 | -0.62 | 0.65 |
| Armenia | 0.66 | 0.06 | 0.66 | 0.12 |  | Iraq | -1.03 | 0.19 | -1.02 | 0.34 |
| Australia | 0.52 | 0.76 | 0.35 | 0.75 |  | Ireland | 0.45 | 0.71 | 0.35 | 0.69 |
| Austria | 1.02 | 0.85 | 1.22 | 0.91 |  | Israel | 0.46 | 0.76 | 0.55 | 0.75 |
| Azerbaijan | -0.76 | 0.39 | -0.28 | 0.32 |  | Italy | -0.55 | 1.00 | -0.31 | 1.00 |
| Bangladesh | 0.18 | 0.19 | 0.18 | 0.34 |  | Japan | 0.82 | 0.85 | 0.90 | 0.91 |
| Belarus | 1.48 | 0.06 | 1.48 | 0.12 |  | Jordan | -1.16 | 0.28 | -1.16 | 0.50 |
| Belgium | 1.15 | 0.39 | 1.40 | 0.32 |  | Kazakhstan | -0.21 | 0.37 | -0.61 | 0.43 |
| Bolivia | -0.17 | 0.37 | -0.11 | 0.43 |  | Kenya | -0.28 | 0.22 | -0.29 | 0.38 |
| Bosnia | -0.98 | 0.53 | -0.51 | 0.54 |  | Kuwait | -0.91 | 0.23 | -1.21 | 0.21 |
| Botswana | 0.64 | 0.13 | 0.64 | 0.22 |  | Kyrgyzstan | 0.86 | 0.06 | 0.86 | 0.12 |
| Brazil | -0.33 | 0.43 | -0.29 | 0.55 |  | Latvia | 0.98 | 0.06 | 0.98 | 0.12 |
| Bulgaria | -0.06 | 0.15 | 0.00 | 0.28 |  | Lebanon | 1.11 | 0.33 | 0.45 | 0.20 |
| Burkina Faso | -0.73 | 0.06 | -0.73 | 0.12 |  | Lithuania | -0.44 | 0.53 | 0.22 | 0.54 |
| Cambodia | -0.32 | 0.13 | -0.32 | 0.22 |  | Luxembourg | 0.38 | 0.39 | 0.01 | 0.32 |
| Cameroon | -1.15 | 0.13 | -1.15 | 0.22 |  | Macedonia Rep | 0.69 | 0.06 | 0.69 | 0.12 |
| Canada | 0.75 | 0.85 | 0.93 | 0.91 |  | Malawi | -0.11 | 0.13 | -0.11 | 0.22 |
| Chile | -0.73 | 0.53 | -0.83 | 0.54 |  | Malaysia | -0.19 | 0.63 | 0.59 | 0.53 |
| China | 0.12 | 1.00 | 0.25 | 1.00 |  | Mali | -1.04 | 0.06 | -1.04 | 0.12 |
| Colombia | -0.66 | 0.76 | -0.84 | 0.75 |  | Malta | 0.08 | 0.06 | 0.08 | 0.12 |
| Costa Rica | -0.06 | 0.45 | -0.20 | 0.59 |  | Mexico | -0.49 | 0.85 | -0.56 | 0.91 |
| Croatia | -0.24 | 0.53 | -0.07 | 0.54 |  | Moldova | -0.17 | 0.53 | 0.27 | 0.54 |
| Czech Rep | 1.03 | 0.85 | 0.47 | 0.91 |  | Montenegro | 1.25 | 0.06 | 1.25 | 0.12 |
| Denmark | 0.98 | 0.63 | 0.86 | 0.53 |  | Morocco | -0.99 | 0.43 | -1.08 | 0.55 |
| Dominican Rep | -1.33 | 0.06 | -1.33 | 0.12 |  | Namibia | -0.95 | 0.23 | -0.70 | 0.21 |
| Ecuador | -0.51 | 0.23 | -0.17 | 0.21 |  | Netherlands | 1.35 | 0.85 | 1.53 | 0.91 |
| Egypt | -0.67 | 0.43 | -0.72 | 0.55 |  | New Zealand | -0.27 | 0.63 | -0.79 | 0.53 |
| El Salvador | -0.84 | 0.38 | -0.83 | 0.49 |  | Nicaragua | -1.65 | 0.13 | -1.65 | 0.22 |
| Estonia | -0.13 | 0.53 | 0.68 | 0.54 |  | Nigeria | -0.97 | 0.76 | -1.01 | 0.75 |
| Finland | 1.27 | 0.76 | 1.02 | 0.75 |  | Norway | 0.91 | 0.39 | 0.57 | 0.32 |
| France | 0.06 | 0.85 | 0.29 | 0.91 |  | Pakistan | -0.09 | 0.19 | -0.07 | 0.34 |
| Georgia | -0.92 | 0.76 | -1.21 | 0.75 |  | Peru | -0.45 | 0.19 | -0.48 | 0.34 |
| Germany | 0.99 | 1.00 | 1.33 | 1.00 |  | Philippines | -0.01 | 0.43 | 0.23 | 0.55 |
| Ghana | -0.37 | 0.19 | -0.47 | 0.34 |  | Poland | -0.27 | 0.85 | -0.07 | 0.91 |
| Greece | -0.62 | 0.85 | -0.69 | 0.91 |  | Portugal | -0.20 | 0.76 | -0.46 | 0.75 |

| Country/region | UP10 | weights | UP6 | weights |  | Country/region | UP10 | weights | UP6 | weights |
| --- | --- | --- | --- | --- | --- | --- | --- | --- | --- | --- |
| Puerto Rico | -1.87 | 0.06 | -1.87 | 0.12 |  | Syria | -1.56 | 0.09 | -1.56 | 0.16 |
| Qatar | -0.43 | 0.23 | -0.08 | 0.21 |  | Taiwan | 0.52 | 0.71 | 0.61 | 0.69 |
| Romania | -0.82 | 0.61 | -0.54 | 0.70 |  | Tanzania | -1.16 | 0.53 | -1.26 | 0.54 |
| Russia | -0.74 | 0.91 | -0.67 | 0.84 |  | Thailand | -0.24 | 0.76 | -0.60 | 0.75 |
| Rwanda | -1.47 | 0.19 | -1.44 | 0.34 |  | Trinidad and Tobago | -1.35 | 0.06 | -1.35 | 0.12 |
| Saudi Arabia | 0.26 | 0.19 | 0.21 | 0.34 |  | Turkey | -0.14 | 0.76 | -0.06 | 0.75 |
| Serbia | -0.16 | 0.19 | -0.13 | 0.34 |  | Uganda | -0.75 | 0.19 | -0.76 | 0.34 |
| Singapore | 1.22 | 0.53 | 1.55 | 0.58 |  | UK | 0.79 | 0.85 | 0.87 | 0.91 |
| Slovakia | 1.30 | 0.06 | 1.30 | 0.12 |  | Ukraine | 0.19 | 0.19 | 0.31 | 0.34 |
| Slovenia | 0.07 | 0.63 | 0.00 | 0.53 |  | United Arab Emirates | -0.24 | 0.13 | -0.24 | 0.22 |
| South Africa | -0.03 | 0.19 | -0.06 | 0.34 |  | Uruguay | -0.79 | 0.06 | -0.79 | 0.12 |
| South Korea | 0.52 | 1.00 | 0.67 | 1.00 |  | USA | 0.64 | 0.85 | 0.67 | 0.91 |
| Spain | -0.07 | 0.91 | -0.32 | 0.84 |  | Venezuela | -0.82 | 0.43 | -0.90 | 0.55 |
| Sri Lanka | -0.26 | 0.13 | -0.26 | 0.22 |  | Vietnam | -0.23 | 0.53 | 0.02 | 0.54 |
| Suriname | 0.03 | 0.13 | 0.03 | 0.22 |  | Zambia | -0.62 | 0.29 | -0.50 | 0.33 |
| Sweden | 1.36 | 0.85 | 1.44 | 0.91 |  | Zimbabwe | -0.68 | 0.43 | -0.56 | 0.55 |
| Switzerland | 1.27 | 0.85 | 1.62 | 0.91 |  |  |  |  |  |  |

**Table B. List of variables and their respective sources.**

| Variable | Source |
| --- | --- |
| Equity risk premium | Excess return of stocks over bonds, as estimated by  (Fernandez P, de Apellániz E, F Acín J. Survey: Market Risk Premium and Risk-Free Rate used for 81 countries in 2020. SSRN. 2015 March. doi: 10.2139/ssrn.3560869.)  (Fernández P, Aguirreamalloa J, Acín IF. Required Market Risk Premium among Countries in 2012. JFDS. 2015 Dec 1;1(1):42-54. doi: 10.1016/j.jfds.2015.07.003.) |
| Average years in school | Expected years of schooling is a component of the Education index from  (Human Development Data (1990-2018). [Internet]. Human Development Reports [cited 2020 July 31]. Available from: http://hdr.undp.org/en/data#) |
| Human Development Index | United Nations Development Programme from  (Human Development Data (1990-2018). [Internet]. Human Development Reports [cited 2020 July 31]. Available from: http://hdr.undp.org/en/data#) |
| Credit rating | Average long-term foreign currency credit rating for sovereign bonds as reported by Standard & Poor’s, Fitch and Moodys (Sovereign credit ratings by country: the latest data. [Internet]. The Global Economy. [cited 2020 July 31]. Available from: https://www.theglobaleconomy.com/rankings/credit_rating/) |
| Gasoline price | Measured by the ratio of the gasoline price to the world average. (Esty DC, Levy M, Srebotnjak T, De Sherbinin A. Environmental sustainability index: Benchmarking national environmental stewardship. New Haven: YCELP. 2005 Feb:47-60.) |
| Resilience index | A measurement of enterprise resilience to disruptive events, calculated by FM Global. (FM Global [Internet]. FM Global Resilience Index. [cited 2019 May]. Available from: https://www.fmglobal.com/research-and-resources/tools-and-resources/resilienceindex/explore-the-data/?) |
| GDP/capita | Gross domestic product as taken from World Bank |
| Interest Rate | Central bank announcements (Interest Rate. [Internet] Trading Economics. [cited 2020 July 31]. Available from: https://tradingeconomics.com/country-list/interest-rate) |
| Credit spreads on government bonds | Spreads are measured with respect to the US (World Government Bonds – Current Spreads. [Internet]. World Government Bonds. [cited 2020 July 31]. Available from: http://www.worldgovernmentbonds.com/spread-historical-data/) |
